# Self-Declared Physical Activity Levels and Self-Reported Physical Fitness in a Sample of Italian Adolescents during the COVID-19 Pandemic

**DOI:** 10.3390/ejihpe12060049

**Published:** 2022-06-18

**Authors:** Alessandro Gatti, Lorenzo Pugliese, Vittoria Carnevale Pellino, Marco Del Bianco, Matteo Vandoni, Nicola Lovecchio

**Affiliations:** 1 Laboratory of Adapted Motor Activity (LAMA), Department of Public Health, Experimental Medicine and Forensic Science, University of Pavia, 27100 Pavia, Italy; alessandro.gatti08@universitadipavia.it (A.G.); vittoria.carnevalepellino@unipv.it (V.C.P.); 2 Department of Public Health, Experimental Medicine and Forensic Science, University of Pavia, 27100 Pavia, Italy; lorenzo.pugliese@unipv.it (L.P.); marco.delbianco@unipv.it (M.D.B.); 3 Department of Industrial Engineering, University of Rome Tor Vergata, 00133 Rome, Italy; 4 Department of Human and Social Science, University of Bergamo, 24127 Bergamo, Italy; nicola.lovecchio@unibg.it

**Keywords:** COVID-19, self-perception, physical fitness, physical activity level, self-reported physical fitness

## Abstract

Only 20% of children worldwide reach the suggested physical activity (PA) levels, and the COVID-19 restrictions seemed to have worsened this situation. In addition, physical fitness (PF) is a crucial marker of health and combined with PA could predict future health status. The aim of this study was to compare reported PA and PF levels in a sample of Italian adolescents. We administered the International PA Questionnaire and International Fitness Enjoyment Scale to 208 adolescents aged 16.0 ± 1.5 *(N* = 166 females, 16 ± 2.0 years) recruited from a high school in the province of Milan (Italy). The majority of the subjects were “Minimally active” but reported adequate PF levels. In particular, subjects who reported a “Very good” PF perception, had a lower PA level. The misperception of reported PA and PF from our sample could reduce the future PA level in adolescents and lead to a negative spiral of disengagement in PA. These findings should lead to more attention on and improvements to PA promotion in the adolescent population after the restrictions caused by the COVID-19 pandemic.

## 1. Introduction

SARS-CoV-2 or COVID-19 is a virus that causes severe damage to the respiratory system and rapidly spread all around the world. For these reasons, authorities implemented several measures to lower infection rates, such as isolation and lockdowns, closure of training and sports facilities, and the “stay at home” campaign. Even if these measures have been shown to be effective, they also had negative repercussions on physical and mental health [1,2,3,4,5]. In fact, a lot of research has shown an increased state of depression, anxiety, and stress during the quarantine and lockdown periods, both in adults [1] and adolescents [6]. Moreover, using a self-administered questionnaire, several studies reported a reduction in the weekly amount of physical activity (PA) and an increase in sedentary behaviors during the COVID-19 restrictions [7,8,9]. The World Health Organization recommends at least 60 min per day of moderate PA and vigorous PA at least two times per week to achieve the benefits of PA. Despite the clear benefits, only 20% of children worldwide reach the amount of PA suggested [10], and the COVID-19 restrictions seemed to have worsened this situation [7]. In fact, as reported by Pietrobelli et al. [11], children in Italy drastically decreased their PA time and increased the time spent in sedentary activities, such as using electronic devices and sleeping. In addition, Bronikowska et al. [12] and Rodríguez-Larrad et al. [13] showed that COVID-19 confinement reduced the PA levels and increased sedentary behavior. Another important marker of health that has been directly associated with PA is physical fitness (PF) (defined as the ability to perform PA [14,15]), which was divided into different macroareas, such as muscular strength [16] (MS), cardiorespiratory fitness [17] (CRF), and speed—agility [18,19] (SA). PF and its dimension have been associated with several health outcomes; for example, MS and SA are associated with improved bone health and a reduced risk of hypertension and type 2 diabetes [20,21,22], while CRF is related to a reduced risk of developing cancer [23], cardiovascular morbidity and mortality [24], improved mental health [25], and reduction of all-cause mortality [26]. A simple way to measure PF was proposed in a study conducted by Ortega et al. [27], and it consists of a self-administered questionnaire called the International Fitness Scale (IFIS). In this study, Ortega et al. [27] reported that the IFIS is a valid method to measure the self-reported PF (SRPF) and showed a correlation with objectively measured PF. A study conducted by Crocker et al. [28] showed that the self-perceived PF is strongly related to the amount of PA objectively measured and to the amount of self-declared PA. Moreover, before the COVID-19 pandemic, a study conducted by Cartagena et al. [29] on Peruvian adolescents showed a high direct correlation between the SRPF, measured by the IFIS, and self-declared PA. Indeed, according to the biopsychological rationale of the questionnaires, there should be a strong relationship between what is perceived and the real practice of the subject. Unfortunately, the COVID-19 restrictions seemed to have an influence on the SRPF as well; in fact, as reported by Makizako et al. [30], there has been a significant decline in SRPF. Since many studies reported that the COVID-19 restrictions changed many aspects of our daily routines, our study aims to understand if the COVID-19 restrictions changed the self-perception about PF in adolescents by comparing the amount of weekly PA and SRPF; in particular, we take into account the period following the first lockdown in Italy (May–June 2020).

## 2. Materials and Methods

### 2.1. Participants

We conducted a study on 208 adolescents aged 16.0 ± 1.5 (166 females and 42 males) recruited from a high school in the province of Milan (Italy). The subjects were asked to participate in the study during curricular Physical Education (PE) classes. The curricular teacher and a sports specialist administered both the International Fitness Enjoyment Scale (IFIS) questionnaire and the IPAQ short form, both in validated Italian form. To participate, all students must have been healthy and eligible to perform PE (as certified upon medical examination), aged between 14 and 18 years, and have Italian language proficiency. Exclusion criteria were a noncomprehension of Italian language, orthopedic injuries, and absolute contraindications to the PA practice. Anthropometric and descriptive characteristics of all the samples are shown in Table 1. The study protocol was approved by the teachers’ board and by the president of the internal council of the school in accordance with the Helsinki Declaration of 1975, as revised in 2018 [31]. All the participants were free to withdraw their participation at any time. Written informed consent was obtained from the parents or legal guardians during the official enrollment, while verbal assent was obtained from the children before participation. No extra credits were gained by the students.

### 2.2. International Physical Activity Questionnaire Short Form (IPAQ-SF)

The IPAQ-SF is a valid and reliable (Cronbach’s alpha = 0.60) [32] questionnaire to assess PA levels in adolescents and adults aged older than 15 years. This questionnaire investigates three specific types of activity: walking, moderate-intensity activities, and vigorous-intensity activities. In particular, the rating (measured in days per week) and duration (time per day) were collected separately for each specific type of activity. The items were structured to provide separate scores on walking and moderate-intensity and vigorous-intensity activity as well as a combined total score to describe the overall level of the performed activity. The total score requires the summation of the duration (in minutes) and frequency (days) of walking and moderate-intensity and vigorous-intensity activity.

After the definition of the minutes spent on the three activities for a week according to the IPAQ-SF guidelines [33], a multiplicative process was carried out. In particular, each type of activity was defined in metabolic equivalents (METs) using the value of: 3.3 for walking, 4 for moderate, and 8 for vigorous activities by Ainsworth et al. [34]. The sum of METs was then calculated to define the amount of weekly PA.

### 2.3. International Fitness Scale (IFIS)

The IFIS is a self-reported, simple, and short fitness scale previously validated in many different countries and languages [35]. The IFIS consists of a 5-point Likert scale (from 1 very poor to 5 very good) with questions focused on five macroareas of fitness: general fitness, cardiorespiratory fitness (CRF, capacity of the circulatory and respiratory systems to supply oxygen to skeletal muscles during physical activity), muscular strength (MS, ability of a muscle to exert a maximal or near maximal force against an object), speed–agility (SA, ability to perform a movement within a short time and to rapidly and accurately change the position/direction of the entire body in response to a stimulus), and flexibility. The IFIS score was proven to be a predictive measure for the objectively measured PF by Ortega et al. [27]. For school-aged students, the IFIS had high validity and moderate-to-good reliability (average weighted Kappa: 0.70 and 0.59; Cronbach’s alpha ranged from 78 to 89 [35]).

### 2.4. Procedure

Both questionnaires were administrated in the same week. The IFIS questionnaire was administrated one day before the PE lesson, while to avoid any influence from the IFIS responses and the PA during the PE lesson, the IPAQ-SF was administered one hour before the PE lesson.

The compilation was anonymous, and to match the results of the two questionnaires, a unique code was used, corresponding to the school enrollment protocol number. The study was conducted between the 20th of May and the 10th of June 2020. The study protocol, including each feature of the experimental design, was approved by the ethical boards of the schools in accord with the World Medical Association Declaration of Helsinki as revised in 2018. According to the informed consent, all participants were free to withdraw their participation at any time, a prior, signed by parents or legal guardians.

### 2.5. Statistical Analysis

**Figure 1:**
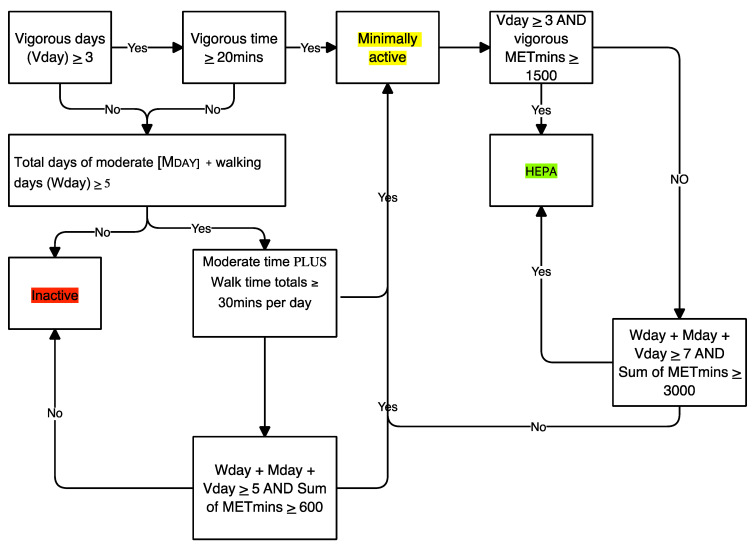
Flowchart algorithm for the analysis of IPAQ short form.

To analyze the IPAQ-SF data, as suggested by the IPAQ-SF international guidelines [33], we classified adolescents into three different levels (inactive, minimally active, and health-enhancing physical activity (HEPA)), based on the continuous measure (METs-week), the frequency, and volume of weekly PA, as reported in Figure 1. The IPAQ categories and IFIS items are represented as response frequencies. Finally, to quantify the relationships between the variables, the Spearman correlation coefficient was applied. Correlation values were interpreted following Cohen’s classification thresholds [36]: 0.30 to 0.59, moderate; 0.60 to 0.79, high; and ≥0.80, excellent. The significance level was set at a *p*-value of <0.05. Statistical analyses were performed using The Jamovi project (2021). Jamovi Version 1.6 for Mac [Computer Software], Sydney, Australia; retrieved from https://www.jamovi.org (accessed on 20 May 2022 ).

## 3. Results

Data for the levels of SRPA are shown in Table 2. For all the groups, the “Inactive” adolescents were slightly more than the “HEPA”, while more than one in two of the adolescents reported being “Minimally active”.

Data for the levels of SRPF are shown in Table 3. For all the IFIS items, except for flexibility, there were more adolescents who perceived these as “Poor” compared to those who perceived them as “Good”.

**Figure 2:**
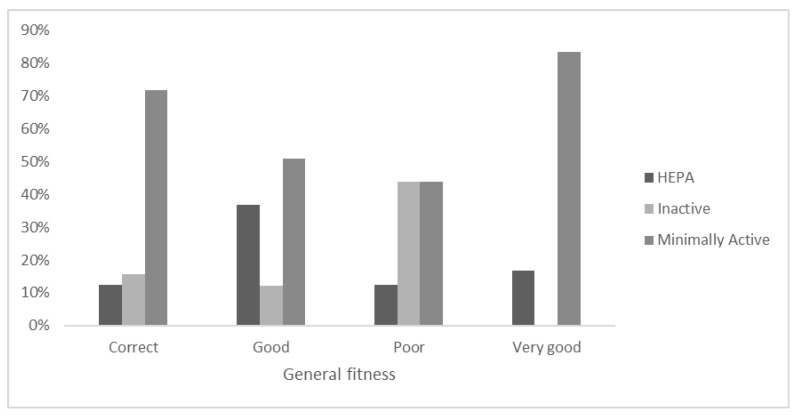
Stratification of PA level categories within the self-declaration of General fitness.

Within the self-perception of General fitness (Figure 2), the category “Very Good” revealed that 83% of the sample was minimally inactive. Within the “Good” self-declaration sample, 50% of the students were minimally active considering the PA level from the IPAQ-SF.

The 12.3% of those who reported “Good” General fitness were Inactive from the evaluation of the PA level.

**Figure 3:**
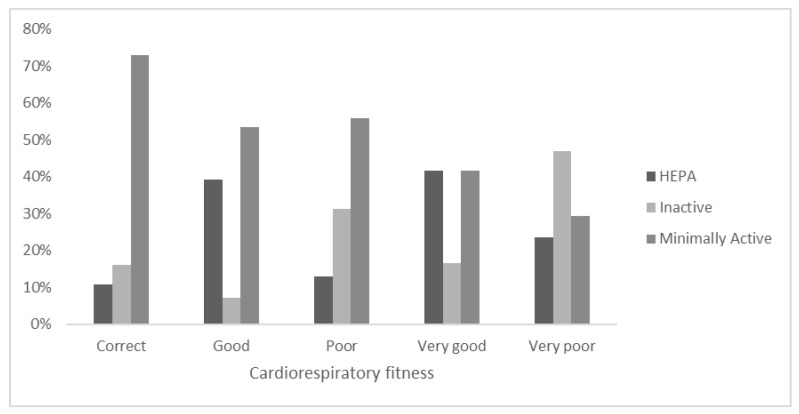
Stratification of PA level categories within the self-declaration of Cardiorespiratory fitness.

In the self-perception of CRF (Figure 3), the category “Very good” revealed that more than 15% of the adolescents were “Inactive”, while 41.7% were “Minimally active”. For those who declared a “Very poor CRF”, 23.5% were HEPA according to the PA level. Within the “Good” self-declaration sample, more than half was “Minimally active”, and a small portion (12.2%) was “Inactive”.

**Figure 4:**
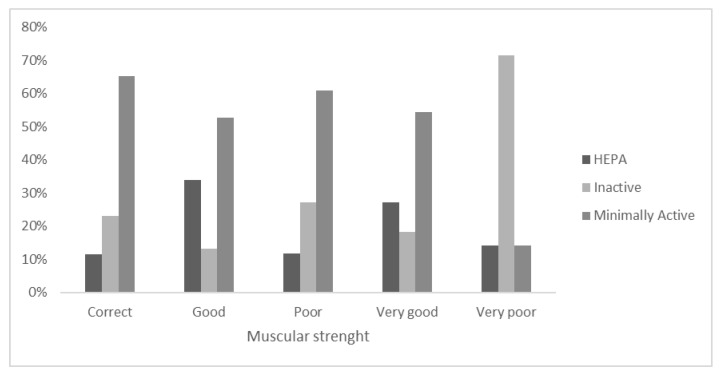
Stratification of PA level categories within the self-declaration of Muscular strength.

Within the self-perception of MS (Figure 4), in the category “Very good”, 18.2% of the adolescents were “Inactive”, while 54.5% were “Minimally active”. For the “Very poor” category, 14.3% were “HEPA” according to the PA level. In the self-perceived Poor category for the MS sample, 11.9% were HEPA, and 61% were Minimally active. In the self-declared Correct category for MS, slightly less than three quarters (65.4%) were “Minimally active”. For the “Good” category, more than half of the adolescents (52.8%) were “Minimally active”.

**Figure 5:**
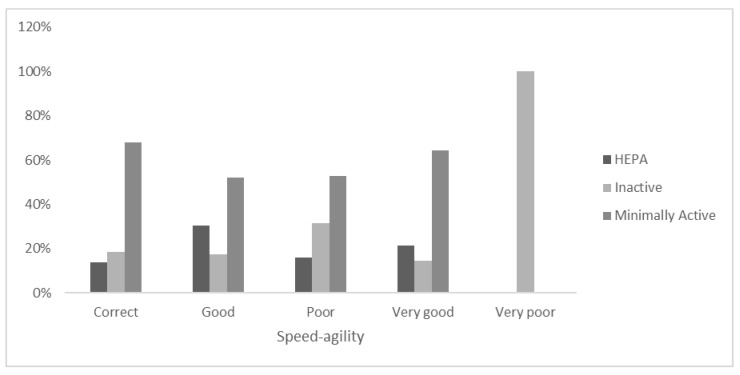
Stratification of PA level categories within the self-declaration of Speed–agility.

In the self-perceived SA sample (Figure 5), for the “Very good” category, 14.3% of the adolescents were “Inactive”, while 64.3% were “Minimally active”. For the self-declared “Very poor” category for SA, no adolescents were “HEPA” or “Minimally active”. Within the self-perceived “Poor” sample, 15.8% were “HEPA”, and 52.6% “Minimally active” according to the PA level evaluation. A total of 17.4% of the adolescents who reported “Good” SA were “Inactive”, while 50% were “Minimally active”. In the self-declared “Correct” sample, 67.8% were “Minimally active”, while 18.4% were “Inactive”.

**Figure 6:**
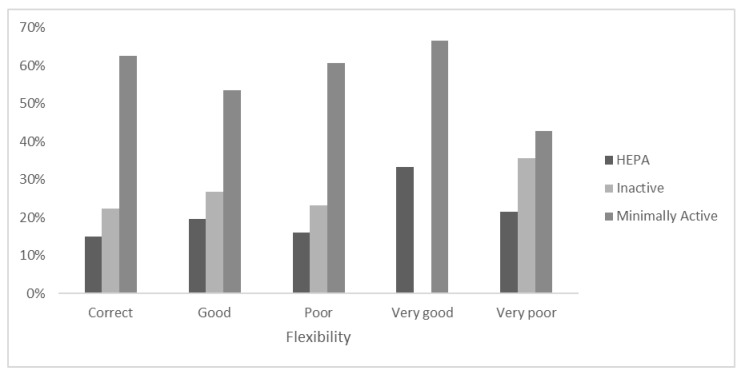
Stratification of PA level categories within the self-declaration of Flexibility.

In the Flexibility category (Figure 6), 66.7% of the adolescents who perceived their flexibility as “Very good” were “Minimally active”. In the “Very poor” self-perceived category, 21.4% were “HEPA”, while in the self-declared “Poor” category, 16.1% were “HEPA”, and 60.7% were “Minimally active”. Within the “Good” and “Correct” self-reported category, 26.8% and 22.4%, respectively, of the sample were “Inactive”, while 53.6% and 62.7% were “Minimally active”.

In the General fitness, CRF, MS, and SA categories, there was a significant direct correlation with the level of SRPA, while for Flexibility there was no significant correlation. However, the correlation coefficient showed a low-to-moderate relationship according to Cohen’s classification between the IFIS items and the PA levels (Table 4).

## 4. Discussion

To the best of the authors’ knowledge, this is one of the first Italian studies that investigated the effects of the COVID-19 restrictions on the self-perception of PF in adolescents. The results of our study highlight that the adolescents were not correctly perceiving PF compared to the amount of declared weekly PA. In fact, for each IFIS item, adolescents incongruously perceived PF compared to weekly PA levels. For example, 83.3% of those who declared to perceive their general fitness as “Very good” were “Minimally active” during the week, showing a high misperception of their abilities. From our results, this phenomenon seems to be more consistent in the adolescents who declared to perceive PF as “Very good”. In fact, for all the PF items perceived as “Very good”, more than half of the samples were “Inactive” during the week. The comparison between self-declared the PA level and SRPF showed that even if there is a significant correlation between four of the five IFIS items and the IPAQ-SF, the correlation coefficient is classified “low to moderate”, which confirms that many adolescents from our sample underestimated or overestimated their PF compared to their PA level. From the current literature, no other studies analyzed the trends of the SRPF during the COVID-19 pandemic in adolescents. Only Makizako et al. [30] showed a significant decline for adults in the SRPF in the Japanese population during the COVID-19 restrictions. Before the COVID-19 restrictions, several studies showed that adolescents correctly perceived their PF; therefore the adolescents who declared higher levels of PA also reported a high level of SRPF [37,38]. In fact, a study conducted by Malete et al. [39] demonstrated that physical self-perception is directly related to the amount of PA in adolescents. The importance of studying the trends of SRPF in adolescents is highlighted by several studies that proved a relationship between the SRPF and adherence to PA. A model proposed by Stodden et al. [40], in fact, indicated the SRPF as a strong carrier for promoting positive adherence to PA. Moreover, longitudinal research conducted by Inchley et al. [41] confirmed the model conceived by Stodden, showing that a high SRPF is correlated with a high level of future PA in adolescents. In addition, Timo et al. [42] showed that the self-perception of PF in adolescents has an effect on subsequent adherence to physical activity; the higher this perception, the more likely a high level of physical activity will be. Considering the results of the previous study, the “misperception” of SRPF reported from our sample and caused by the COVID-19 restrictions could alter this model, also reducing the future PA level in adolescents, leading to a negative spiral of disengagement in PA with low SRPF, less PA level, poor health-related physical fitness, and a final decline in health status. Therefore, our results highlight the role of physical education teachers and coaches to enhance adolescent perception of performance with stimulating routines in order to ultimately promote an active lifestyle.

Another interesting result that we reported is the very low number of adolescents who declared to be HEPA (18%), while more than 20% of the adolescents reported being “inactive”. No great differences were observed between boys and girls for the PA levels. In accordance with our results, several studies showed that the COVID-19 restrictions negatively impacted the PA levels in adolescents. For example, Gallè et al. [43] showed an increment in sedentary behavior and a decrease in PA in Italian adolescents. The same situation was reported by Ruiz-Roso [44], who showed an increment in sedentary behavior and in ultraprocessed food consumption. A reduced level of PA has been shown to be related to an increase in the risk for anxiety, depression [45], and several diseases, such as cardiovascular disease, type 2 diabetes, and obesity [46]. To counteract the negative effects of the restrictive measures adopted during the COVID-19 emergency, some new learning approaches and methodologies were introduced [47,48] and should be implemented, i.e., web home-based PA, active video gaming, and physical education in online learning [49].

This study has some limitations mainly due to the sample size, the type of performed evaluations (questionnaires instead of PF tests), and lack of consideration for the biological maturation, which could influence the perception of PF. Unfortunately, restriction from the first lockdown were severe because of social distancing (stay at home and the stopping of physical activities and exercise) imposed by the government also in the school setting. For these reasons, we were unable to recruit a larger sample to directly perform a test session and to assess the biological maturation of the students.

## 5. Conclusions

In conclusion, our results show that the COVID-19 restrictions had an impact on the self-perception of PF and on the PA levels in Italian adolescents. Future studies should analyze the correlation between the SRPF and the objectively measured PF to confirm if the COVID-19 restrictions really impacted the SRPF. These findings should lead health policy makers to promote PA in the adolescent population and to enhance specific programs of exercise in school and everyday settings to combat a prospective worsening of health status.

## Figures and Tables

**Table 1:** Anthropometric and descriptive characteristics of all the samples and of boys and girls.

	N	Age (Years)	Height (m)	Weight (kg)	BMI (kg/m^2^)
**All the** **samples**	208	16.5 ± 1.5	1.6 ± 0.2	57.1 ± 10.1	20.6 ± 2.9
**Boys**	42	16.4 ± 3.0	1.7 ± 0.3	65.2 ± 9.3	20.8 ± 2.6
**Girls**	166	16.4 ± 2.0	1.6 ± 0.2	54.5 ± 10.5	20.4 ± 3.5

N = numerosity; BMI = body mass index; m = meters; kg = kilograms.

**Table 2:** Level of self-reported PA for all the samples and of boys and girls.

	Inactive		Minimally Active		HEPA	
	N	%	N	%	N	%
**All the sample**	48	23.1%	122	58.6%	38	18.3%
**Boys**	9	21.4%	27	64.3%	6	14.3%
**Girls**	39	23.5%	95	57.2%	32	19.3%

**Table 3:** Level of self-reported physical fitness for all the samples.

	Very Poor		Poor		Correct		Good		Very Good	
	N	%	N	%	N	%	N	%	N	%
**General** **fitness**	0	0.0%	64	30.8%	89	42.8%	49	23.6%	6	2.9%
**Cardiorespiratory fitness**	17	8.1%	77	37.0%	74	35.6%	28	13.5%	12	5.8%
**Muscular strength**	7	3.3%	59	28.4%	78	37.5%	53	25.5%	11	5.3%
**Speed–agility**	4	1.9%	57	27.4%	87	41.8%	46	22.1%	14	6.7%
**Flexibility**	14	6.7%	56	26.9%	67	32.2%	56	26.9%	15	7.2%

**Table 4:** Correlation between the level of self-declared PA and self-reported PF.

	IPAQ-SF	
	Correlation Coefficient	*p*-Value
**General fitness**	0.323 ***	<0.001
**Cardiorespiratory fitness**	0.258 ***	<0.001
**Muscular strength**	0.239 ***	<0.001
**Speed–agility**	0.205 **	0.003
**Flexibility**	0.089	0.203

Note: ** *p* < 0.01; *** *p* < 0.001.

## Data Availability

Not applicable.
